# Public-access defibrillation and favorable neurological outcome after out-of-hospital cardiac arrest during the COVID-19 pandemic in Japan

**DOI:** 10.1186/s13054-022-04220-9

**Published:** 2022-10-31

**Authors:** Tasuku Matsuyama, Kosuke Kiyohara, Tetsuhisa Kitamura, Chika Nishiyama, Takeyuki Kiguchi, Taku Iwami

**Affiliations:** 1 Department of Emergency Medicine, Kyoto Prefectural University of Medicine, Kamigyo-Ku, Kyoto, 602-8566 Japan; 2 Departments of Food Science, Otsuma Women’s University, Tokyo, Japan; 3 Division of Environmental Medicine and Population Services, Department of Social and Environmental Medicine, Graduate School of Medicine, Osaka University, Osaka, Japan; 4 Department of Critical Care Nursing, Kyoto University Graduate School of Human Health Science, Kyoto, Japan; 5 Kyoto University Health Service, Kyoto, Japan

**Keywords:** Out-of-hospital cardiac arrest, Public-access defibrillation, COVID-19

## Abstract

**Background:**

Early public-access defibrillation (PAD) effectively improves the outcomes of out-of-hospital cardiac arrests (OHCA), but several strategies implemented to prevent the spread of coronavirus disease 2019 (COVID-19) could decrease the availability of PAD and worsen outcomes after OHCA. Previous studies have reported conflicting findings, and there is a paucity of nationwide observations. This study aims to investigate the impact of COVID-19 on PAD and OHCA outcomes using a nationwide OHCA registry in Japan, where PAD is well-documented.

**Methods:**

This secondary analysis of the All-Japan Utstein Registry, a prospective population-based nationwide registry of OHCA patients, included patients aged ≥ 18 years with bystander-witnessed OHCA and an initial shockable rhythm who were transported to medical facilities between January 1, 2005, and December 31, 2020. The analytical parameters of this study were the proportion of patients who underwent PAD and patients with one-month survival with favorable neurological outcomes, defined as a cerebral performance category score of 1 or 2. We compared the data between 2019 and 2020 using a multivariable logistic regression analysis.

**Results:**

During the study period, 1,930,273 OHCA patients were registered; of these, 78,302 were eligible for the analysis. Before the COVID-19 pandemic, the proportion of OHCA patients who underwent PAD and demonstrated favorable neurological outcomes increased gradually from 2005 to 2019 (P for trend < 0.001). The proportion of patient who had PAD were 17.7% (876/4959) in 2019 and 15.1% (735/4869) in 2020, respectively. The proportion of patient who displayed favorable neurological outcomes were 25.1% (1245/4959) in 2019 and 22.8% (1109/4869) in 2020, respectively. After adjusting for potential confounders, a significant reduction in the proportion of PAD was observed compared to that in 2019 (adjusted odds ratio [AOR], 0.86; 95% confidence interval [CI], 0.76–0.97), while no significant reduction was observed in favorable neurological outcomes (AOR, 0.97; 95% CI 0.87–1.07).

**Conclusion:**

The proportion of PAD clearly decreased in 2020, probably due to the COVID-19 pandemic in Japan. In contrast, no significant reduction was observed in favorable neurological outcomes.

## Background

Out-of-hospital cardiac arrest (OHCA) is an important public health issue in industrialized countries, affecting more than 120,000 individuals in Japan [1–3]. Neurological outcomes after OHCA have improved gradually owing to recent advances in care, but patient mortality remains high [1–3].

Early public-access defibrillation (PAD) effectively improves outcomes after OHCA [4, 5]. Despite the proven effectiveness of rapid PAD by bystanders, public-access AEDs are rarely used worldwide, and only approximately 5% of bystander-witnessed OHCA with cardiac origin received the benefit of PAD in Japan [3, 4]. In addition, several strategies implemented to prevent the spread of coronavirus disease 2019 (COVID-19), such as lockdown, travel restrictions, and physical distancing, could decrease the availability of PAD and worsen outcomes after OHCA during the COVID-19 era. Previous studies have reported conflicting findings in this regard, and there is a paucity of nationwide observations [6]. Therefore, we aimed to evaluate the impact of COVID-19 on PAD and OHCA outcomes using a nationwide OHCA registry in Japan, where PAD is well-documented [4].

## Methods

### Study design and setting

This is a secondary analysis of the All-Japan Utstein Registry, a prospective population-based nationwide registry of OHCA patients based on the internationally standardized style [7]. Details of the registry and emergency medical service (EMS) system in Japan have been previously described [4, 8]. This study included patients aged ≥ 18 years with bystander-witnessed OHCA and an initial shockable rhythm who were transported to medical facilities between January 1, 2005, and December 31, 2020. Since July 2004, citizens have been legally permitted to use automated external defibrillators (AEDs). In Japan, approximately two million citizens have participated in cardiopulmonary resuscitation (CPR) programs that include trainings on chest compression, mouth-to-mouth ventilation, and AED use [4]. In Japan, the first COVID-19 patient was documented on January 15, 2020. A total of 239,192 COVID-19 cases were confirmed, and 3501 COVID-19 patients had died by the end of 2020 [9]. There were three waves of the COVID-19 pandemic in Japan in 2020. From April 7 to May 25, 2020, a state of emergency was declared because of the first rapid spread of COVID-19, which was a type of lockdown in Japan, and mobility was strongly restricted [9].

### EMS organization in Japan

Details of the EMS system in Japan have been reported elsewhere [4, 8]. In brief, the EMS is provided by local fire stations where an ambulance from a nearby fire station is dispatched when a 119 call is received. Among EMS personnel, emergency life-saving technicians (ELSTs), who are highly trained pre-hospital emergency care providers, are permitted to provide an intravenous line, adjunct airway, and a semi-automated external defibrillator. Typically, there are three emergency providers, including at least one ELST in an ambulance. Specially trained ELSTs are also permitted to perform tracheal intubation and administer intravenous epinephrine. EMS providers perform CPR according to the Japanese guidelines for CPR [10]. Do-not-resuscitate orders are generally not accepted in pre-hospital settings in Japan. EMS providers are not allowed to terminate resuscitation. Therefore, most patients with OHCA treated by EMS personnel are transported to hospitals and enrolled in the All-Japan Utstein Projects. In addition to standard precautions, EMS personnel must wear N95 face masks and isolation gowns when contacting patients with OHCA during the COVID-19 pandemic. Since April 24, 2020, paramedics have been encouraged to insert a supraglottic airway instead of endotracheal intubation.

### Data collection and quality control

The All-Japan Utstein Registry prospectively collected data according to the Utstein-style reporting guidelines for cardiac arrest [7]. The data included patient age, patient sex, origin of arrests (cardiac or non-cardiac), type of bystander (family member or other), first documented cardiac rhythm, time course of resuscitation, type of bystander-initiated CPR, dispatcher instruction, delivery or non-delivery of public-access AED shocks, advanced airway management, epinephrine administration, and outcomes including 1-month survival rates. The time of receipt of an emergency call, initial contact with patients, initiation of CPR, defibrillation performed by EMS personnel, and hospital arrival were recorded using the clock of each EMS system. When bystanders delivered shock using public-access AEDs, the first recorded rhythm was regarded as shockable rhythm ventricular fibrillation (VF) or pulseless ventricular tachycardia (VT). EMS personnel completed the data forms in cooperation with the treating physicians. Data were uploaded to the All-Japan Utstein Registry database server and logically checked using a computer system. When data were incomplete, the Fire and Disaster Management Agency requested the fire stations to supply the missing details.

### Outcome measures

The outcomes of this study were PAD and one-month survival with favorable neurological outcomes, defined as a cerebral performance category score of 1 or 2 [11].

### Ethics statements

This study was approved by the ethics committee of Kyoto Prefectural University of Medicine (number: ERB-C-1164), which waived the requirement for written informed consent due to the retrospective nature of the study. Personal identifiers were excluded from Utstein records.

### Statistical analysis

The trend in the proportion of PAD and favorable neurological outcomes was assessed using the Cochran–Armitage trend test. Next, we compared the outcomes between 2019 and 2020 using a multivariable logistic regression analysis. The covariates included in the analysis for PAD were patient age, patient sex, family bystander witness, dispatcher CPR instruction, and EMS response time [4, 8]. We also documented PAD, pre-hospital advanced airway management, and pre-hospital adrenaline administration. Furthermore, to evaluate the impact of “state of emergency,” we compared outcomes between 2019 and 2020 according to “the state of emergency (April 7 to May 25, 2020)” and “outside the state of emergency” using the same methods mentioned above [4, 8]. All statistical analyses were performed using SPSS statistical package version 25.0 J (IBM Corp., Armonk, NY, USA).

## Results

**Fig. 1:**
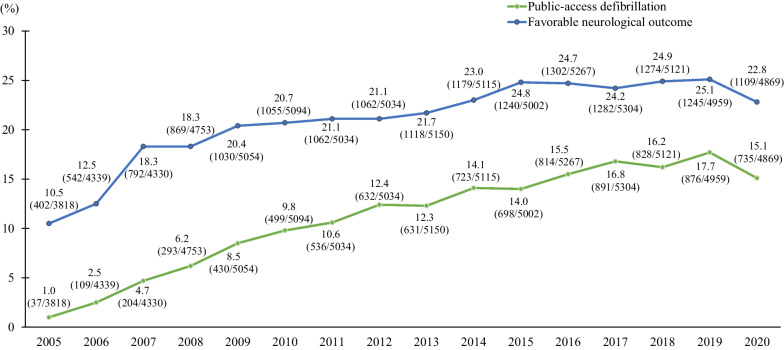
Trends of public-access defibrillation and favorable neurological outcome after out-of-hospital cardiac arrest in Japan

During the study period, 1,930,273 OHCA patients were registered; of these, 78,302 were eligible for the analysis. Before the COVID-19 pandemic, the proportion of patients with OHCA who had PAD and displayed favorable neurological outcomes increased gradually from 2005 to 2019 (P for trend < 0.001) (Fig. 1).

The proportion of patient who had PAD were 17.7% (876/4959) in 2019 and 15.1% (735/4869) in 2020, respectively. The proportion of patient who displayed favorable neurological outcomes were 25.1% (1245/4959) in 2019 and 22.8% (1109/4869) in 2020, respectively. After adjusting for potential confounders, a significant reduction in the proportion of patients who had PAD was observed compared to that in 2019 (adjusted odds ratio [AOR], 0.86; 95% confidence interval [CI], 0.76–0.97), while no significant reduction was observed in favorable neurological outcomes (AOR, 0.97; 95% CI 0.87–1.07) (Table 1). The proportion of patients who had PAD significantly decreased during the state of emergency in 2020 compared to the same period in 2019 (20.9% [133/635] in 2019 vs 9.9% [58/587] in 2020, AOR, 0.65; 95% CI 0.48–0.89), while there was no significant change in favorable neurological outcomes during this period (25.2% [160/635] in 2019 vs 24.5% [144/587] in 2020, AOR: 1.19, 95% CI: 0.88–1.60) (Table 1).

**Table 1:** Public-access defibrillation and favorable neurological outcome according to the period

	2019	2020	The same period as State of emergency in 2019 (April 7 to May 25)	State of emergency in 2020 (April 7 to May 25)	Other period in 2019	Other period in 2020
	(n = 4959)	(n = 4869)	(n = 635)	(n = 587)	(n = 4324)	(n = 4282)
Public-Access Defibrillation	876 (17.7)	735 (15.1)	133 (20.9)	58 (9.9)	743 (17.2)	677 (15.8)
Crude OR (95% CI)	Reference	0.83 (0.74–0.92)	Reference	0.41 (0.30–0.58)	Reference	0.91 (0.81–1.01)
Adjusted OR (95% CI)*	Reference	0.86 (0.76–0.97)	Reference	0.45 (0.31–0.66)	Reference	0.93 (0.82–1.06)
Favorable neurological outcome	1245 (25.1)	1109 (22.8)	160 (25.2)	144 (24.5)	1085 (25.1)	965 (22.5)
Crude OR (95% CI)	Reference	0.88 (0.80–0.97)	Reference	0.97 (0.74–1.25)	Reference	0.87 (0.79–0.96)
Adjusted OR (95% CI)†	Reference	0.97 (0.87–1.07)	Reference	1.19 (0.88–1.60)	Reference	0.94(0.84–1.05)

*Adjusted for age, sex, witnessed by family, dispatcher instruction, and EMS response time

^†^Adjusted for age, sex, witnessed by family, dispatcher instruction, public-access defibrillation, prehospital advanced airway management, prehospital adrenaline administration, and EMS response time

OHCA indicates out-of-hospital cardiac arrest; OR, odds ratio; CI, confidence interval; EMS, emergency medicine personnel

## Discussion

These findings demonstrate that the proportion of patients who had PAD decreased by 2020, turning from the previous upward trend. Similar to a previous study, a marked decrease was observed during the state of emergency [12]. This result was probably due to travel restrictions and physical distancing. The reason for the lack of significant change in favorable neurological outcomes remains unknown but might be attributable to the lack of sufficient sample size to detect statistical significance.

Rapid PAD makes 2–3 folds increase in favorable neurological outcomes after OHCA [4, 5]. The actual prevalence of PAD has been very low, even before the COVID-19 era. We observed an even lower proportion of patients who underwent PAD during the COVID-19 era. Currently, the International Liaison Committee on Resuscitation (ILCOR) suggests the use of AED, although they do not have evidence of whether defibrillation generates aerosols [13]. To avoid further reduction in the use of PAD, it may be suggested to advocate that PAD does not increase the risk of COVID-19 infection in rescuers from patients with cardiac arrest. Therefore, ILCOR should make more efforts to recommend rapid AED use.

This study had several inherent limitations. First, the Utstein Style–based registry did not provide details about patients’ activities of daily living or medical conditions before the arrest. Second, information on the actual incidence of COVID-19 in each region during the study period was unavailable. Third, the residual confounders may have been similar to those in other observational studies.

In conclusion, the proportion of patients who had PAD clearly decreased in 2020, probably due to the COVID-19 pandemic in Japan. In particular, a marked decrease was observed during the state of emergency. Conversely, no significant reduction was observed in favorable neurological outcomes.

## Data Availability

Please contact the author for data requests.

## Abbreviations

AED: Automated external defibrillators
CI: Confidence interval
CPR: Cardiopulmonary resuscitation
ELST: Emergency life-saving technicians
EMS: Emergency medical service
ILCOR: International Liaison Committee on Resuscitation
OHCA: Out-of-hospital cardiac arrest
PAD: Public-access defibrillation
VF: Ventricular fibrillation
VT: Ventricular tachycardia

## References

[1] Merchant RM, Topjian AA, Panchal AR, Cheng A, Aziz K, Berg KM (2020). Part 1: executive summary: 2020 American heart association guidelines for cardiopulmonary resuscitation and emergency cardiovascular care. Circulation.

[2] Perkins GD, Graesner JT, Semeraro F, Olasveengen T, Soar J, Lott C (2021). European resuscitation council guidelines 2021: executive summary. Resuscitation.

[3] Fire and Disaster Management Agency. Report on a study on social system development to improve survival from emergency cardiovascular disease (in Japanese). https://www.fdma.go.jp/publication/#rescue Accessed September 1, 2022

[4] Kitamura T, Kiyohara K, Sakai T, Matsuyama T, Hatakeyama T, Shimamoto T (2016). Public-access defibrillation and out-of-hospital cardiac arrest in Japan. N Engl J Med.

[5] Investigators TPADT (2004). Public-access defibrillation and survival after out-of-hospital cardiac arrest. N Engl J Med.

[6] Bielski K, Szarpak A, Jaguszewski MJ, Kopiec T, Smereka J, Gasecka A (2021). The influence of COVID-19 on out-hospital cardiac arrest survival outcomes: an updated systematic review and meta-analysis. J Clin Med.

[7] Perkins GD, Jacobs IG, Nadkarni VM, Berg RA, Bhanji F, Biarent D (2015). Cardiac arrest and cardiopulmonary resuscitation outcome reports: update of the Utstein Resuscitation Registry Templates for Out-of-Hospital Cardiac Arrest: a statement for healthcare professionals from a task force of the International Liaison Committee on Resuscitation (American Heart Association, European Resuscitation Council, Australian and New Zealand Council on Resuscitation, Heart and Stroke Foundation of Canada, InterAmerican Heart Foundation, Resuscitation Council of Southern Africa, Resuscitation Council of Asia); and the American Heart Association Emergency Cardiovascular Care Committee and the Council on Cardiopulmonary, Critical Care. Perioper Resuscit Circ.

[8] Iwami T, Kitamura T, Kiyohara K, Kawamura T (2015). Dissemination of chest compression-only cardiopulmonary resuscitation and survival after out-of-hospital cardiac arrest. Circulation.

[9] Ministry of Health, Labour and welfare. Visualizing the data: information on COVID-19 infections https://covid19.mhlw.go.jp/extensions/public/en/index.html. Accessed August 25, 2022.

[10] Japan Resuscitation Council. 2015 Japanese Guidelines for Emergency Care and Cardiopulmonary Resuscitation. TokyoJapan: Igaku-Shoin, 2016

[11] Becker LB, Aufderheide TP, Geocadin RG, Callaway CW, Lazar RM, Donnino MW (2011). Primary outcomes for resuscitation science studies: a consensus statement from the American Heart Association. Circulation.

[12] Marijon E, Karam N, Jost D, Perrot D, Frattini B, Derkenne C (2020). Out-of-hospital cardiac arrest during the COVID-19 pandemic in Paris, France: a population-based, observational study. Lancet Public Health.

[13] International Liaison Committee on Resuscitation. Consensus on Science with Treatment Recommendations (CoSTR): COVID-19 infection risk to rescuers from patients in cardiac arrest: Systematic Review. https://costr.ilcor.org/document/covid-19-infection-risk-to-rescuers-from-patients-in-cardiac-arrest-systematic-review

